# Deletion of the Viral Thymidine Kinase in a Meq-Deleted Recombinant Marek’s Disease Virus Reduces Lymphoid Atrophy but Is Less Protective

**DOI:** 10.3390/microorganisms10010007

**Published:** 2021-12-22

**Authors:** Steven J. Conrad, Eniope B. Oluwayinka, Mohammad Heidari, Jody K. Mays, John R. Dunn

**Affiliations:** 1 United States Department of Agriculture, Agricultural Research Service, U.S. National Poultry Research Center, Avian Disease and Oncology Laboratory, East Lansing, MI 48823, USA; steven.conrad@usda.gov (S.J.C.); mohammad.heidari@usda.gov (M.H.); jody.mays@usda.gov (J.K.M.); 2 Department of Veterinary Medicine, Federal University of Agriculture Abeokuta, Abeokuta 111101, Nigeria; jacobseb@funaab.edu.ng

**Keywords:** Marek’s disease, MD, Marek’s disease virus, MDV, live-attenuated virus, thymidine kinase, tk, Meq

## Abstract

Marek’s disease (MD) is a ubiquitous disease of domesticated chickens and its etiologic agent is the *Gallid alphaherpesvirus* 2 (GaHV-2), also known as Marek’s disease virus (MDV). MD is currently controlled by vaccination using live attenuated strains of MDV (e.g., CVI988/Rispens), non-pathogenic serotypes of MDV (GaHV-3), or non-pathogenic strains of the related *Melagrid alphaherpesvirus* 1 (MeHV-1). One attractive strategy for the production of new vaccine strains is a recombinant MDV attenuated by the deletion of the major viral oncogene *meq*. However, *meq*-deleted variants of MDV cause atrophy of the bursa and thymus in maternal antibody-negative chickens, and the resulting immunosuppression makes them unsuitable. Herein we detail our attempt to mitigate the lymphoid atrophy caused by *meq*-deleted MDV by further attenuation of the virus through ablation of the viral thymidine kinase (*tk*) gene. We demonstrate that ablation of the viral *tk* from the *meq*-deleted virus rMd5B40/Δ*meq* resulted in a virus attenuated for replication in vitro and which spared chickens from atrophy of the lymphoid organs in vivo. When the rMd5B40/Δ*meq*/Δ*tk*/GFP was used as a vaccine it was protective against challenge with the vv+MDV strain 686, but the protection was less than that provided by the CVI988/Rispens vaccine.

## 1. Introduction

The *Gallid alphaherpesvirus* 2 (genus *Mardivirus*, subfamily *Alphaherpesvirinae*, family *Herpesviridae*) also known as Marek’s disease virus (MDV) is the etiologic agent of Marek’s disease (MD) in chickens [1]. In poultry MDV causes an acute and highly contagious lymphoproliferative disease and the lifecycle of MDV in its host has been extensively characterized and reviewed [2]. According to the “Cornell model” the initial infection occurs when shed desquamated feather follicle epithelial cells hosting MDV are engulfed by macrophages in the lungs of the host [3,4]. Early lytic infection is initially detected in B and T cells (although B cells are dispensable for the infection cycle, see [5]), while later infection is characterized by the amplification of recruited, activated and subsequently transformed CD4^+^ CD25^+^ T cells [6]. Clinical manifestations of MD include inflammation of peripheral nerve tracts resulting in gait abnormalities and paralysis, atrophy of the lymphoid organs (bursa and thymus) with subsequent immunosuppression, and the formation of visceral tumors derived from transformed CD4^+^ T cells [7]. MDV establishes a latent state by approximately 21 days post-infection, during which the MDV genome can be detected integrated into the chromosomes of transformed CD4^+^ CD25^+^ T cells [8]. The resulting life-long infection can be reactivated from latency to a productive lytic state from latently infected and transformed CD4^+^ T cells [9].

Due to MDV’s highly contagious nature, its ability to persist in the rearing environment and the high degree of morbidity and mortality associated with MD it is one of the most significant pathogens of poultry in the United States and worldwide. In commercial flocks, MD is controlled by universal vaccination in ovo. Currently the CVI988/Rispens vaccine is the best available option for control of MD and is most commonly administered alone or in combination with the heterologous virus MeHV-1/HVT (MDV serotype 3) [10]. Broiler chickens are frequently vaccinated with either HVT or with multi-valent preparations consisting of both HVT and SB-1 [11].

All live attenuated virus vaccines used for protection against MD, including the CVI988/Rispens vaccine strain, are non-sterilizing vaccines (sometimes referred to as “leaky” or “imperfect”) [11]. These vaccines prevent MDV-induced tumorigenesis, reduce viral load during subsequent infection with MDV field strains and prevent the development of overt clinical pathology but they do not prevent infection, viral replication in the host, viral shedding, or horizontal transmission of either the vaccine strain or of other MDV field isolates [11,12]. Imperfect vaccination may be an important driver of viral evolution toward more virulent strains [11]. History has demonstrated that over time new and increasingly virulent strains of MDV will emerge and “break” the existing protection [13,14]. The possibility that universal vaccination against MD is driving viral evolution towards more virulent field isolates and MDV’s history of increasing virulence over time both provide impetus to the search for more protective vaccine options [13,15]. An ideal MD vaccine candidate would not only prevent overt pathology from new and more-virulent field isolates of MDV but would also spare the lymphoid organs from atrophy in maternal antibody-negative chickens.

Several groups have attempted to create new and more protective vaccine strains from MDV by ablation of its major viral oncogene, *meq* (so named because of its location on the MDV EcoRI Q fragment, [16]). *meq* is an oncogene unique to MDV and exists in two copies in the MDV genome. For example, in the genome of the very virulent (vv) MDV strain Md5 *meq* exists in two copies, designated MDV005 and MDV076, one each in the internal repeat long (IRL) and the terminal repeat long (TRL) genome segments [17]. Its gene product is the oncoprotein Meq and was initially identified as a basic leucine zipper (bZIP)-like protein with homology to the fos/jun family of nuclear-localized oncogenes [16]. Meq is a nuclear phosphoprotein of 339 amino acids [18], and is able to participate as a dimerization partner with other bZIP region-containing proteins [19]. As a homodimer, Meq functions as a transcriptional repressor and can operate upon both its own promoter sequence and upon the MDV origin of replication [19]. The Meq/Jun heterodimer acts as a transcriptional activator for these same sequences and is also capable of binding to control elements for host genes as well [19]. Both homodimerization and heterodimerization with the Jun oncoprotein are necessary for transformation of host cells [20]. In addition to heterodimerization with the c-Jun transcription factor, Meq has been shown to interact with other cellular proteins, including the histone deacetylases 1 and 2 (HDAC1 and 2) and the multifunctional protein and “guardian of the genome” p53 [21,22]. It is reasonable to expect that additional Meq interaction partners will be identified. MDV mutants lacking the *meq* gene are non-tumorigenic and for this reason several groups have experimented with *meq*-ablated mutants as live attenuated vaccine candidates [23,24,25]. Although *meq*-ablated MDV recombinants are generally protective in these same studies, they have consistently caused atrophy of the thymus and bursa when used as vaccine strains in chickens negative for anti-MDV maternally derived antibodies. We reasoned (as have others) that further attenuation of a *meq*-deleted very virulent pathotype (vv) strain of MDV, the rMd5B40 strain (Md5 harboring the B40 bacterial artificial chromosome B40, see [26]) might render it protective yet benign with respect to atrophy of the thymus and bursa. In these experiments we examined an MDV strain, rMD5B40, ablated for the viral thymidine kinase (*tk*) as a possible solution to the problem of lymphoid atrophy caused by the rMd5B40/Δ*meq* virus.

In the alphaherpesviruses the viral thymidine kinase (*tk*) is a non-essential virulence factor and has frequently been a target of ablation for the purpose of creating live attenuated virus vaccines (e.g., [27]; reviewed in [28]). Although the entire sequence of the herpesvirus *tk* is not highly conserved across the *Herpesviridae*, there are six highly conserved subregions within the gene that are conserved [28]. In MDV (as well as in herpes simplex viruses 1 and 2, [29]) the thymidine kinase is encoded by the UL23 gene [17]. In the alphaherpesviruses the viral *tk* is a polynucleotide kinase able to phosphorylate both deoxythymidine and deoxycytidine [28], but at present the full substrate range of the MDV *tk* has not been characterized. Ablation of the viral *tk* from other alphaherpesviruses (e.g., see [27]) is a common strategy for viral attenuation, and viral attenuation by this method has also been demonstrated in a variety of other double-stranded DNA viruses in vitro (e.g., the Asfarviridae, [30]; the Poxviridae [31]). Ablation of the viral *tk* has also been shown to reduce virulence in vivo in animal models of herpesvirus virulence [28]. In our experiments we used the recombinant MDV rMd5B40 strain from which both copies of *meq* have been deleted [32] as a backbone vector for further modification. We hypothesized that ablation of the MDV *tk* gene would result in a virus attenuated for replication efficiency and that the reduced replication would spare the bursa and thymus from damage and atrophy. We ablated the viral *tk* by insertion of sequence encoding the fluorescent reporter mTag green fluorescent protein (mTagGFP), a variant of GFP with enhanced stability and a faster maturation time. The recombinant viruses used in this study, rMd5B40/Δ*meq* and rMd5B40/Δ*meq*/Δ*tk*/GFP, were characterized for their replication potential both in vitro and in vivo, and for their vaccinal protective potential in vivo against the very virulent plus (vv+) strain MDV 686.

## 2. Materials and Methods

### 2.1. Research Animals

The animals used in these experiments were F_1_ crosses between two specific pathogen-free (SPF) MDV-susceptible chicken lines, 15I_5_ males (MD resistant) and 7_1_ females (MD susceptible), all of which were negative for maternally derived antibodies against MDV. Chickens were housed in Horsfall–Bauer isolators and were monitored daily. All bird experiments were approved by the ADOL Institutional Animal Care and Use Committee; approval no. 2020-07.

### 2.2. Cells and Viruses

Virus propagation and titration were done in cultured secondary duck embryonic fibroblast (DEF) cells which were derived from SPF Khaki-Campbell ducks. Cells were cultured in medium composed of equal parts Leibovitz medium (L4386–10 L, SIGMA, St. Louis, MO, USA) and McCoy’s 5A medium (M4892–10 L, SIGMA), supplemented with 10% premium grade heat-inactivated FBS (Atlanta Biologicals, Flowery Branch, GA, USA) except after viral inoculation for titration, when 2% FBS was used.

#### Ablation of the Viral Thymidine Kinase

The starting backbone virus for the construction of the rMd5B40/Δ*meq*/Δ*tk/eGFP* virus was the rMd5B40/Δ*meq* recombinant, which was deleted for both copies of *meq* and has been described previously [26,32]. We used the mTagGFP as a fluorescent reporter in the construction of the rMd5B40/Δ*meq*/Δ*tk/GFP* virus. Using the unique *Bste*II endonuclease recognition sites withing the mTagGFP ORF of the pCMV6-AC-mTagGFP plasmid (Origene catalog number PS100010), the kanamycin ORF was inserted into the mTagGFP ORF and the correct orientation of the insert was verified by sequencing through the mTagGFP ORF (sequencing data not shown). Primers with termini homologous to the TK/UL23 ORF were then used to amplify the modified plasmid, resulting in an mTagGFP ORF interrupted with a kanamycin ORF with termini homologous to the rMd5 *tk* gene. Verification of the insertion and its orientation was done by PCR fragment size (Figure S1). Subsequent to clonal isolation of successful recombinant viruses, two-step red-mediated mutagenesis [33] was used to insert the kanamycin-interrupted mTagGFP sequence into the viral *tk* locus. The sw105 strain of E. coli harboring the pBAD-I-*Sce*I arabinose-inducible *Sce*I homing endonuclease was used to excise the kanamycin ORF, and recombinants in which the kanamycin ORF was successfully excised were selected by negative selection for kanamycin resistance, resulting in a viral mutant in which the functional mTagGFP had replaced the viral *tk* gene. The ablation of the viral *tk* was verified by PCR (Figure S2) and subsequent Sanger sequencing (data not shown). The resulting recombinant viruses were maintained and titrated on monolayers of secondary DEFs. The primer sequences used in the construction of the rMd5B40/Δ*meq*/Δ*tk/GFP* virus used in these experiments are detailed in the Supplementary Table S1. 

### 2.3. Assessment of Recombinant MDVs In Vitro

In vitro replication kinetics of recombinant MDV strains rMd5B40 (wild type), rMd5B40/Δ*meq* (parental) and rMd5B40/Δ*meq*/Δ*tk*/GFP were compared for their replication kinetics. MDV rMd5B40 or each of the recombinant viruses were separately plated on monolayers of secondary DEFs and the degree of viral replication assessed by quantification of MDV glycoprotein B mRNA by quantitative polymerase chain reaction (refer to Section 2.4 for details of the gB/GAPDH qPCR), where chicken GAPDH mRNA quantity was used as a normalizer. Monolayers were inoculated at a multiplicity of infection of 0.1, and data collected at 2, 3 and 5 days post inoculation.

### 2.4. Quantification of Viremia Produced by Recombinant MDVs

At each of two sampling times (day 6 and day 13 post inoculation), ten chickens per treatment were randomly selected for whole blood sampling, from which peripheral blood lymphocytes (PBLs) were isolated. Viral DNA was isolated from PBLs using a Puregene DNA isolation kit (Gentra System, Minneapolis, MN, USA) followed by analysis of viral genome content by a quantitative multiplex real-time polymerase chain reaction (qPCR). MDV glycoprotein B was used as an indicator of viral abundance and GAPDH was used as a normalizer. Glycoprotein B was detected with the primers 5′-CGGTGGCTTTTCTAGGTTCG-3′ (F), 5′-CCAGTGGGTTCAACCGTGA-3′ (R) and the probe 5′FAM-CATTTTCGCGGCGGTTCTAGACGG-3′-BHQ. GAPDH was detected with the primers 5′-CAACGGTGACAGCCATTCCT-3′ (F), 5′-ATGGTCGTTCAGTGCAATGC-3′ (R) and the probe 5′-Vic -CCTTTGATGCGGGTGCT-BHQ-1-3′. For all qPCR reactions the TaqMan Fast Advanced Master Mix (Applied Biosystems) was used. Data is presented as the ratio of MDV glycoprotein B to GAPDH.

### 2.5. Assessment of Lymphoid Atrophy Caused by Recombinant MDVs

Chickens were challenged at day of hatch with 2000 plaque-forming units (pfu) of either the CVI988/Rispens (our negative control for lymphoid atrophy), rMd5B40/Δ*meq*, or rMd5B40/Δ*meq*/Δ*tk*/GFP viruses. Body and lymphoid organ weights were determined at the 6- and 13-day time points and for analysis and the lymphoid organ weights were expressed as a proportion of the total body weight to control for animal-to-animal variation. The reported data were produced in two independent replicate experiments and pooled for analysis.

### 2.6. Assessment of Protection Provided by rMDVs

Chickens were vaccinated at day of hatch with 2000 pfu of the appropriate recombinant MDV strain or the CVI988/Rispens vaccine strain (a highly protective vaccine strain used as a positive control) to assess each recombinant virus for vaccinal protection. At 5 days post-hatch, all groups (except the no vaccination control/negative control) were challenged with 500 pfu of the vv+ strain MDV686. Animals were either euthanized as the humane endpoint was reached or continued to approximately 60 days post-challenge. All animals were necropsied at the time of death to assess tumors and peripheral nerve enlargement, which are diagnostic for the presence of MD. Each group began the experiment with a total of 15 chickens in each HB isolator. The reported data was produced in two replicate experiments and pooled for analysis.

### 2.7. Significance Level and Statistical Analysis of Data

The *p* = 0.05 level was selected as the threshold for statistical significance before experiments were initiated. All statistical analysis and visualization of data was done with GraphPad Prism (v8.0) from GraphPad Software (San Diego, CA, USA, www.graphpad.com). For viral growth kinetics and for comparison of viremias in vivo the Welch’s-corrected unpaired t test was used to compare each time point to the controls. For the weights of animals and lymphoid organs we used the non-parametric Mann--Whitney U test to compare the means of the control and experimental groups. For survival (Kaplan–Meier) curve analysis we used the Mantel–Cox Chi-square test to determine *p* values. The survival and protection studies were done as two independent replicate experiments and the results were pooled for analysis.

## 3. Results

### 3.1. Ablation of the Viral tk from the rMd5B40/Δmeq Virus Attenuates Replication In Vitro

**Figure 1:**
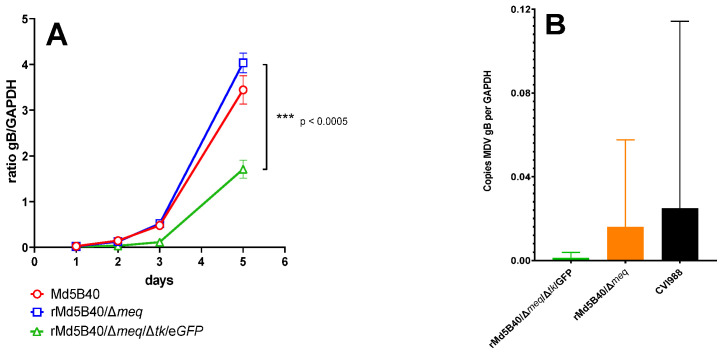
Ablation of the *tk* gene in MDV rMd5B40 attenuates replication both in vitro and in vivo. (**A**) When viral replication was assayed in vitro, the rMd5B40Δmeq/Δ*tk*/eGFP variant accumulated significantly less glycoprotein B transcripts than either the wild-type virus or the rMd5B40/Δ*meq*. (**B**) In vivo, the Δ*tk* recombinant also trended towards less viremia 6 days after inoculation at day-of-hatch, but the differences between these groups and the rMd5B40/Δ*meq* parental were not statistically significant (data not shown). For panel A data were compared using unpaired t-tests, (***) indicates a statistically significant difference between groups. For B, data were compared using ordinary ANOVA analysis.

The wild-type MDV rMd5B40, the rMd5B40/Δ*meq* and the rMd5B40/Δ*meq*/Δ*tk*/GFP were compared for growth kinetics in vitro on monolayers of secondary DEF cells. Figure 1A shows that over the 5-day experiment both the MDV rMd5B40 and the rMd5B40/Δ*meq* viruses accumulated approximately the same quantity of viral glycoprotein B transcripts as measured by qPCR and normalized to the amount of GAPDH mRNA. Significantly less viral glycoprotein B transcript was detected in the rMd5B40/Δ*meq*/Δ*tk*/GFP-inoculated cultures than in the Md5B40 cultures at days 2, 3 and 5. By day 5, the rMd5B40/Δ*meq*/Δ*tk*/GFP virus had accumulated approximately half (*p* < 0.01) of the glycoprotein B message when compared to the rMd5B40 and the rMd5B40/Δ*meq* viruses. Replication of the Δ*tk* recombinants was likewise reduced in vivo. Figure 1B shows that at 6 days post inoculation, when inoculated into day-of-hatch chicks, both of the Δ*tk* recombinants produced less viremia than the CVI988 vaccine strain or the rMd5B40/Δ*meq* recombinant. Unfortunately high group variability in the rMd5B40/Δ*meq* and CVI988 groups prevents us from saying definitively that viral replication in vivo was also attenuated. We therefore suggest that ablation of the MDV *tk* results in a replication-attenuated virus in vitro.

### 3.2. Attenuation of the rMd5B40/Δmeq Virus by Ablation of the Viral tk Spared Chickens from Lymphoid Atrophy

**Figure 2:**
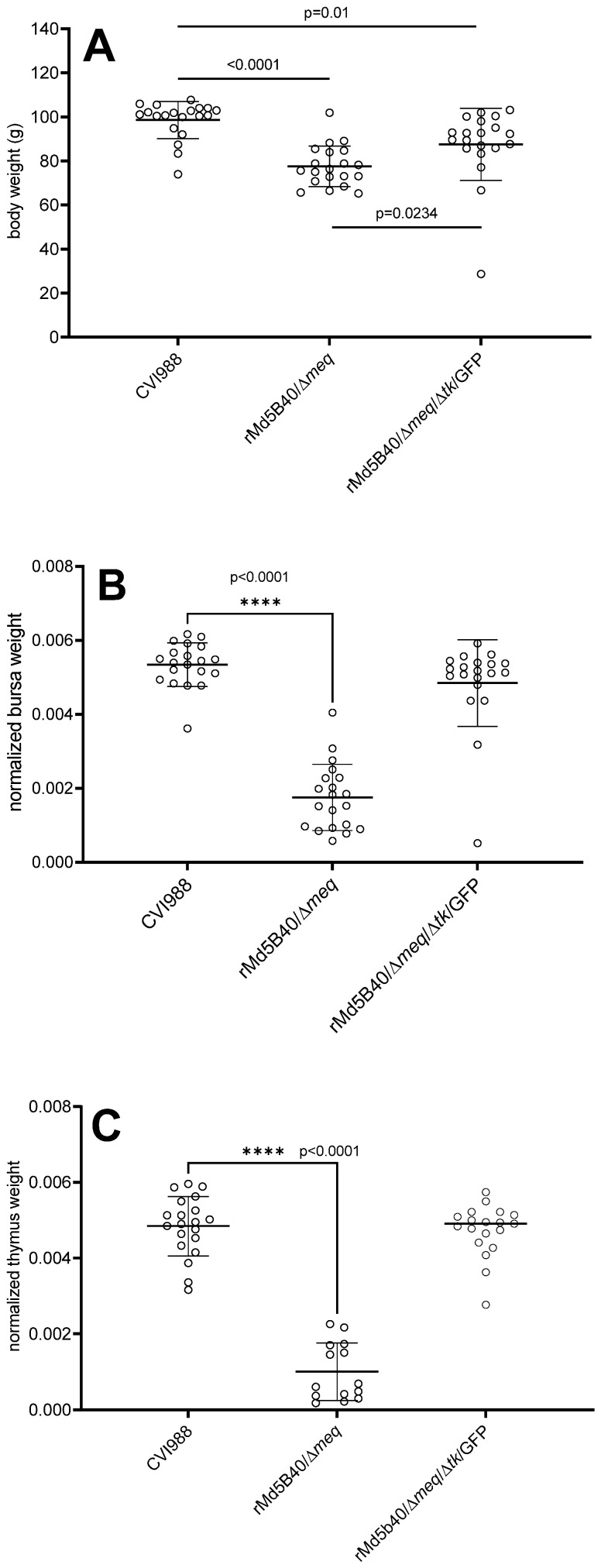
Ablation of the *tk* gene from the rMd5B40/Δ*meq* virus mitigates atrophy of the lymphoid organs. 13-day old chickens (**A**) body weight is significantly reduced with inoculation of the recombinant viruses compared to the CVI988 control, but much less in the *tk*-deleted recombinant. The (**B**) bursa weights and (**C**) thymus weights were normalized to body mass. In each case only the *meq*-ablated recombinant causes detectable atrophy of the lymphoid organs. Each recombinant virus-inoculated group is compared to the CVI988 controls. Data were compared using unpaired t-tests, (****) indicates a statistically significant difference between groups.

The rMd5B40/Δ*meq*/Δ*tk*/GFP virus was compared to both the parental rMd5B40/Δ*meq* virus and the CVI988/Rispens virus with respect to body weight and to determine the degree of lymphoid atrophy produced when used alone in maternal antibody-negative chickens. The CVI988/Rispens is an important comparator because it is the industry-leading vaccine at the time of our study. We examined the 13-day post-inoculation time point because viral replication should be readily detectable yet not reduced by entry into the lytic phase. As shown in Figure 2A–C, by 13 days post-inoculation clear differences among the groups could be discerned. Figure 2A shows that when compared to the CVI988/Rispens-inoculated controls, the rMd5B40/Δ*meq* virus and the rMd5B40/Δ*meq*/Δ*tk*/GFP virus both caused statistically significant reductions in body weight at 13 days post-inoculation. The rMd5B40/Δ*meq* virus caused the most severe reduction in body weight, a 21% reduction (*p* < 0.0001) when compared to the CVI988-vaccinated controls. In the rMd5B40/Δ*meq*/Δ*tk*/GFP-inoculated group the reduction in body weight was less pronounced (11% reduction, *p* = 0.0011). Notably, the rMd5B40/Δ*meq*/Δ*tk*/GFP-inoculated group (mean body weight 87.5 g) experienced less body-weight loss than did the rMd5B40/Δ*meq*-inoculated group (77.6 g), a small but significant (*p* = 0.0234) improvement. As for lymphoid atrophy, Figure 2B,C show that the rMd5B40/Δ*meq*/Δ*tk*/GFP-inoculated group (chicks experienced no significant reduction in lymphoid organ weight, and that only rMd5B40/Δ*meq* virus produced significant atrophy of the thymus (79%, Figure 2B) and bursa (67%, Figure 2C), as anticipated. This data supports our hypothesis that ablation of the *tk* gene from the rMd5B40/Δ*meq* virus curtails its propensity to cause atrophy of the lymphoid organs.

### 3.3. The rMd5B40/Δmeq/Δtk/GFP Viruse Displayed Reduced Protection against MD, against the Development of Visceral Tumors and against Mortality When Challenged with a vv+ MD

Table 1 shows that, compared with the group in which the rMd5B40/Δ*meq* virus was used as a vaccine and subsequently challenged with MDV686 (10% MD+), the rMd5B40/Δ*meq*/Δ*tk*/GFP virus was less protective against the development of the visceral tumors, which are diagnostic of MD (32.1% MD+). The recombinant virus, in which the viral *tk* was ablated, performed significantly worse than did the CVI988 virus and the parental rMd5B40/Δ*meq* virus. 

**Figure 3:**
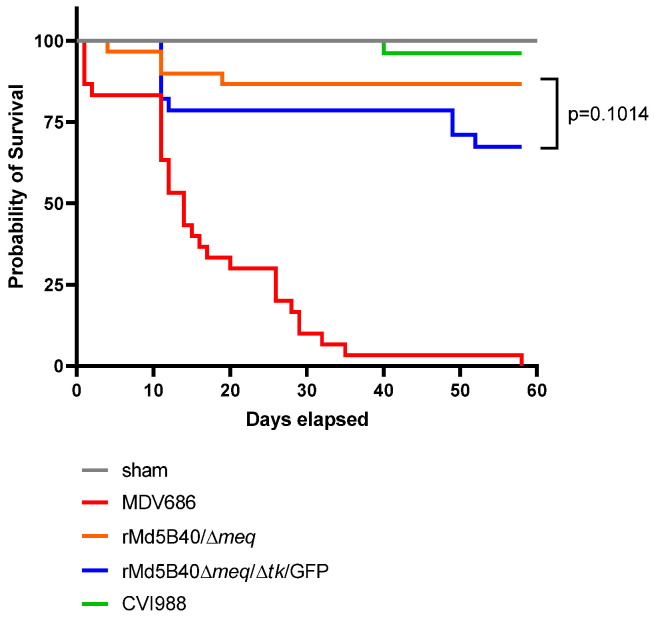
Ablation of the viral *tk* reduces protection against mortality when challenged by a vv+ MDV. The rMd5B40/Δ*meq*/Δ*tk*/GFP (blue line) was not significantly less protective than the parental rMd5B40/Δ*meq* (orange line) in which the *tk* was intact, although survival trended lower in this construct (*p* = 0.1014, Log-rank Mantel-C). As anticipated, the CVI988 vaccine was extremely protective (green line), while unvaccinated animals were highly susceptible to MDV686 in the absence of protection (red line).

Figure 3 illustrates the protective ability of the recombinants. The Kaplan–Meier survival curve shows that in the absence of protection (sham group, gray line), the MDV686 (red line) was indeed lethal over the course of the experiment, with 100% mortality by day 60 post challenge and a median survival time of 14 days. Also, the CVI988/Rispens strain (green line) performed very well, with all but one animal surviving until the end of the study. The best performer of the recombinant viruses was the rMd5B40/Δ*meq* virus (orange line), which was not significantly different from the CVI988/Rispens strain. Although the rMd5B40/Δ*meq* and rMd5B40/Δ*meq*/Δ*tk*/GFP (blue line) were not statistically different (*p* = 0.1014), they were clearly trending towards a significant difference in which the rMd5B40/Δ*meq*/Δ*tk*/GFP recombinant virus provided less protection than did the rMd5B40/Δ*meq* recombinant. For this reason, we must consider the rMd5B40/Δ*meq*/Δ*tk*/GFP virus to be slightly less protective than the rMd5B40/Δ*meq* virus, although the difference did not reach statistical significance.

## 4. Discussion

The rMd5B40/Δ*meq* viruses made and tested by several groups (ours and others, see [23]) have been highly protective as vaccines against vv+ MDV challenge, yet have produced an unacceptable amount of lymphoid organ atrophy. We attempted to attenuate the rMd5B40/Δ*meq* virus by ablation of the viral thymidine kinase. This is a common strategy to attenuate herpesviruses. Our hypothesis was that the lymphoid atrophy caused by the rMd5B40/Δ*meq* recombinant could be alleviated by reducing viral replication in vivo. We engineered a rMd5B40/Δ*meq*/Δ*tk*/GFP virus by ablation of the viral *tk* from rMd5B40/Δ*meq*. The ablation of the viral *tk* resulted in a virus that was significantly attenuated for replication in vitro (see Figure 1). When tested in vivo, the rMd5B40/Δ*meq*/Δ*tk*/GFP recombinant trended towards less viral replication when measured at 6 days post-inoculation, but high variability in the rMd5B40/Δ*meq*-inoculated group precludes the determination of a significant difference between these two groups. When tested in vivo alone (i.e., without a subsequent challenge) we verified that the rMd5B40/Δ*meq* virus did indeed cause lymphoid atrophy, in agreement with previous reports [23]. This data fits well with previous data in which deletion of the viral *tk* has resulted in viruses attenuated for replication [24].

As hypothesized, the ablation of the viral *tk* from the rMd5B40/Δ*meq* recombinant virus did reduce atrophy to the thymus and bursa by an amount sufficient to make it statistically indistinguishable from the CVI988-inoculated controls by 13 days post-inoculation, although there was a very small but significant amount of body weight loss in the rMd5B40/Δ*meq*/Δ*tk*/GFP group. This loss was significantly smaller than was experienced by the rMd5B40/Δ*meq*-inoculated group, however. The *tk*-deleted recombinant rMd5B40/Δ*meq*/Δ*tk*/GFP completely spared the inoculated animals from atrophy of the thymus and bursa. From these results we conclude that ablation of the viral *tk* does indeed attenuate the atrophy caused by the rMd5B40/Δ*meq* virus, although these experiments do not address the mechanism of this reduction. While this is an encouraging finding, for the rMd5B40/Δ*meq*/Δ*tk*/GFP virus to be useful as a potential vaccine it must retain all of the protective capacity of the rMd5B40/Δ*meq* recombinant, which it did not. Table 1 shows that animals vaccinated with the rMd5B40/Δ*meq* recombinant and then challenged with the vv+ MDV686 are highly protected but that animals vaccinated with the rMd5B40/Δ*meq*/Δ*tk*/GFP recombinant and challenged with MDV686 experience visceral tumor formation in the majority of the tested animals (23/32). For protection against mortality, the rMd5B40/Δ*meq*/Δ*tk*/GFP recombinant was indistinguishable from the rMd5B40/Δ*meq* recombinant (*p* = 0.1014) but the data for this group trended towards lower group survival when used as a vaccine against the vv+ pathotype MDV686 strain. From this data, combined with our data which shows that the rMd5B40/Δ*meq*/Δ*tk*/GFP caused a small amount of body weight loss compared with the existing best-in-class vaccine (CVI988/Rispens) it is clear that the most protective of our recombinant viruses, the rMd5B40/Δ*meq*/Δ*tk*/GFP, is not superior in these experiments to the CVI988/Rispens vaccine strain and would, therefore, not be an improvement compared with the currently available CVI988/Rispens.

One possible interpretation of these results is that attenuation of replication in the *meq*-deleted MDV also reduces its protective ability when used as a vaccine, an observation which has been made before [25,34]. Additionally, a very recent paper [35] has demonstrated that ablation of the viral IL-8 analog (vIL8) in a *meq*-deleted background vv+ virus (MDV686BAC) resulted in a vaccine strain that is at least as protective as the CVI988/Rispens strain and that causes no detectable atrophy of either the bursa or thymus when tested alone. The same study also noted a reduction in viral replication of the *meq*- and vIl8-deleted mutant in vivo. This is a clear indication that attenuation mechanisms which merely reduce viral replication in vitro or in vivo cannot be expected to result in superior vaccine strains for protection against MD. Rather, the way forward is likely to be through targeted changes in the immunomodulation capabilities of the virus. If this strategy is borne out in other herpesviruses (and other viruses in general) then its application will allow the production of safe and efficacious vaccines for any number of communicable diseases.

## Figures and Tables

**Table 1:** Ablation of the viral *tk* reduces protection against the development of the visceral tumors diagnostic of MD. Birds were scored for the presence of visceral tumors at necropsy.

Vaccine Strain	Challenge Strain	MD+/Total
none	MDV686	25/30
CVI988/Rispens	MDV686	01/30
rMd5B40/Δ*meq*	MDV686	03/30
rMd5B40/Δ*me*q/*tk*/GFP	MDV686	23/32

## Data Availability

The data presented in this study are available within this paper.

## Supplementary Materials

The following supporting information can be downloaded at: https://www.mdpi.com/article/10.3390/microorganisms10010007/s1, Figure S1: Gel electrophoresis of isolates screened for confirmation. Figure S2: Verification of the insertion and orientation of the GFP marker into the *tk* gene. Table S1: Primers used.
