# Supplementary figures and images for: Deletion of the Viral Thymidine Kinase in a Meq-Deleted Recombinant Marek’s Disease Virus Reduces Lymphoid Atrophy but Is Less Protective
Source: Microorganisms. 2021 Dec 22;10(1):7. doi: 10.3390/microorganisms10010007 (PMC8779792; doi:10.3390/microorganisms10010007)

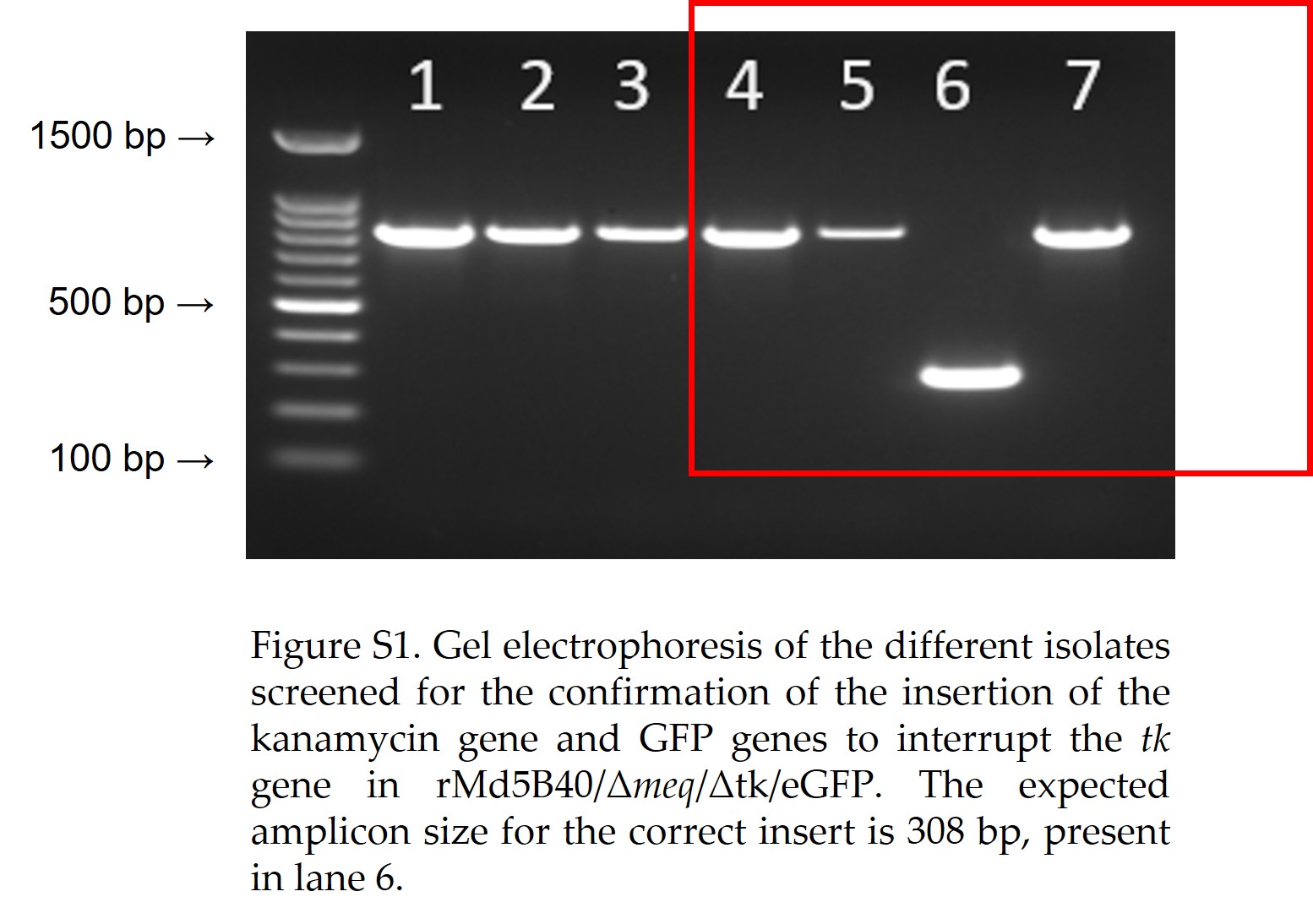

Supplement: Supplementary file 1 [file microorganisms-10-00007-s001.zip › Supplementary files/Figure S1.jpg]

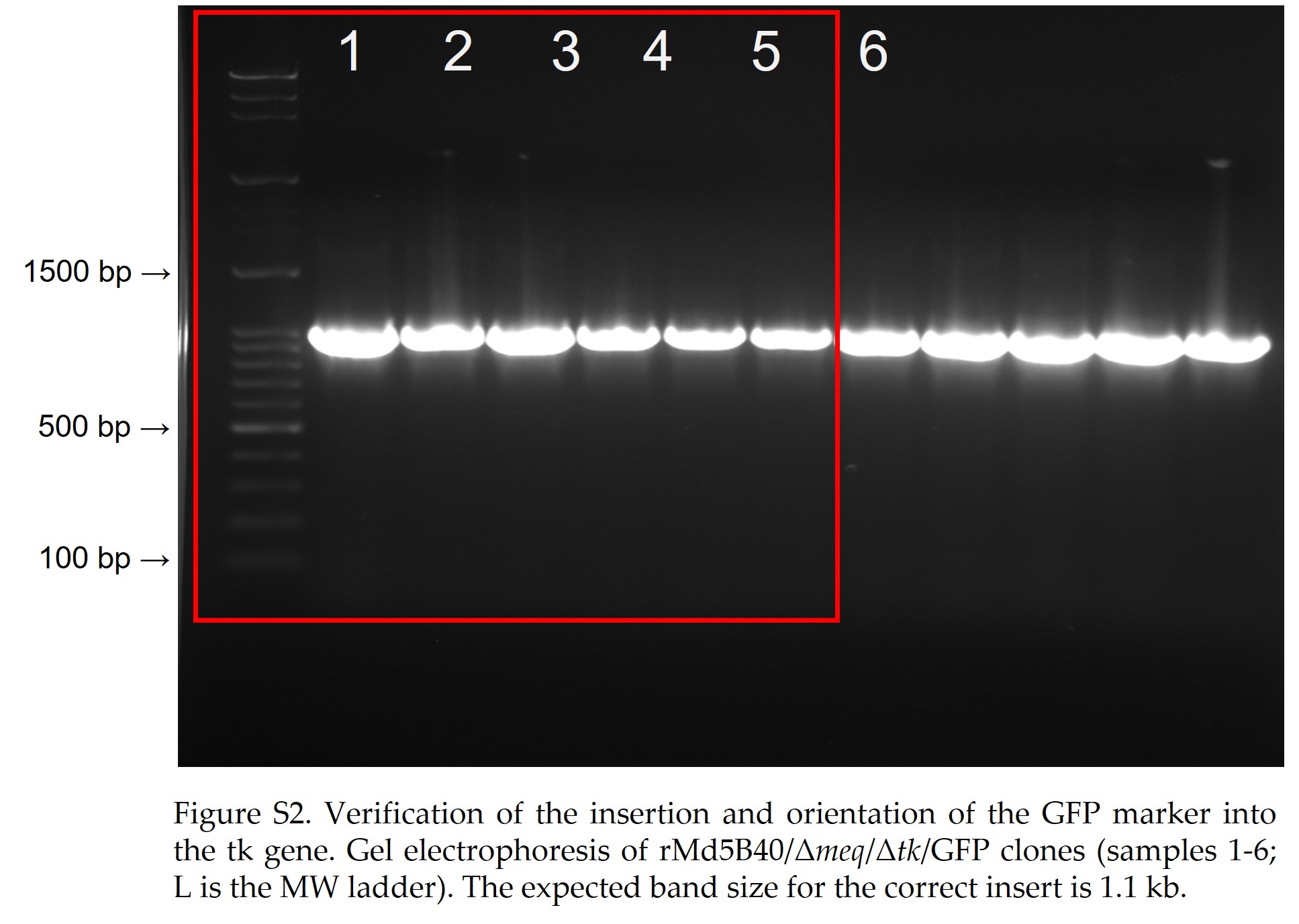

Supplement: Supplementary file 1 [file microorganisms-10-00007-s001.zip › Supplementary files/Figure S2.jpg]
